# Supplementary material for: Passive accumulation of alkaloids in inconspicuously colored frogs refines the evolutionary paradigm of acquired chemical defenses
Source: eLife. 2024 Dec 27;13:RP100011. doi: 10.7554/eLife.100011 (PMC11677230; doi:10.7554/eLife.100011)
Supplement: Supplementary file 2. [file elife-100011-supp2.docx]

**Table S2**. A summary of data available on alkaloid detection in “undefended” lineages of poison frogs prior to this study.

| **Species** | **Method** | **Reported Results** | **Our interpretation** | **Rationale if our interpretation is distinct from reported results** | **Reference** |
| --- | --- | --- | --- | --- | --- |
| *Allobates femoralis* | GC-MS | trace amount of a 5,8-indolizidine alkaloid detected in one individual from one of six tested populations | low levels of an alkaloid present in one skin, possibly in others below detection threshold | N/A | [1] |
| *Allobates femoralis* | TLC | no spots detected in 15 individuals | no alkaloids detected, but image of TLC plate not available to verify interpretation | N/A | [2] |
| *Allobates femoralis* | mouse bioassay and predator assay | no difference in mouse behavior between saline and *A. femoralis* injection, and naïve chicks readily ate three frogs | skin secretion does not contain [enough] compounds that irritate mice or deter chicks | N/A | [3] |
| *Allobates femoralis* | mouse bioassay | difference in mouse behavior between saline and *A. femoralis* injection | skin extracts contain compounds that irritate mice, but unclear if those compounds are lipophilic alkaloids, and others have speculated that irritant effects are due to the benzocaine used for euthanasia [4] | N/A | [5] |
| *Allobates femoralis* | GC-MS | no alkaloids detected in two individuals | no alkaloids detected, but we cannot rule out the occurrence of lipophilic alkaloids below detection thresholds or not in the Daly database | N/A | [4] |
| *Allobates femoralis* | GC-MS | no alkaloids detected in one individual | no alkaloids detected, but we cannot rule out the occurrence of lipophilic alkaloids below detection thresholds or not in the Daly database | the study tested ethanol in which whole animals were soaked after being collected | [6] |
| *Allobates femoralis* | feeding assay (alkaloid-dusted fruit flies) followed by GC-MS | a captive-bred individual had detectable sparteine in skin after a month | the single captive-bred individual tested accumulated provisioned sparteine | N/A | [7] |
| *Allobates femoralis* | oral administration assay (alkaloids dissolved in 50% EtOH) followed by GC-MS | six wild-caught frogs had DHQ in trace quantities (~1% of amount orally administered) in skins, livers, and feces (three individuals), but no HTX **235A** (three individuals) after 14 days | the frogs accumulate DHQ but not HTX **235A** | N/A | [8] |
| *Allobates insperatus* | TLC | no spots detected in 12 individuals | no alkaloids detected, but image of TLC plate not available to verify interpretation | N/A | [2] |
| *Allobates kingsburyi* | TLC | spot observable in three individuals | spot on TLC plate suggests presence of skin metabolites (potentially alkaloids) | the negative control used in this experiment (*H. yasuni*) yielded spots on the TLC plate but was assumed to lack alkaloids, which likely confounded the interpretation of results | [9] |
| *Allobates myersi* | GC-MS | no alkaloids detected in one individual | no alkaloids detected, but we cannot rule out the occurrence of lipophilic alkaloids below detection threshold | N/A | [1] |
| *Allobates sumtuosus* | GC-MS | no alkaloids detected in two individuals | no alkaloids detected, but we cannot rule out the occurrence of lipophilic alkaloids below detection thresholds or not in the Daly database | the study tested ethanol in which whole animals were soaked after being collected | [6] |
| *Allobates talamancae* | either by mouse bioassay or [^3^H]saxitoxin binding assay | no alkaloids detected (likely in one individual) | no evidence of lipophilic alkaloid presence or absence | water-based extraction process would not retain high levels of lipophilic alkaloids, so cannot be used as evidence of presence or absence | [10,11] |
| *Allobates talamancae* | GC-MS | no alkaloids detected in 10 individuals | no alkaloids detected, but we cannot rule out the occurrence of lipophilic alkaloids below detection thresholds or not in the Daly database | the study tested ethanol in which whole animals were soaked after being collected | [6] |
| *Allobates talamancae* | feeding assay (alkaloid-dusted fruit flies) followed by GC-MS | no alkaloids detected in unknown number of individuals | no evidence for alkaloid accumulation after five weeks | N/A | [12] |
| *Allobates talamancae* | unknown (possibly GC-MS) | no alkaloids detected in unknown number of wild-caught individuals | no alkaloids detected, but we cannot rule out the occurrence of lipophilic alkaloids below detection threshold | N/A | unpub. data reported in [13] |
| *Allobates zaparo* | TLC | either no or weak (at most) spots detected in 20 individuals | spot on TLC plate suggests presence of skin metabolites (potentially alkaloids) | Darst et al. [2] bin this species into a non-toxic category despite presence of light spots | [2] |
| *Allobates zaparo* | mouse bioassay and predator assay | no difference in mouse behavior between saline and *A. zaparo* injection, and naïve chicks readily ate six frogs | skin secretion does not contain [enough] compounds that irritate mice or deter chicks | N/A | [14] |
| *Allobates zaparo* | mouse bioassay | no difference in mouse behavior between saline and *A. zaparo* injection, using skin samples from five individuals | skin secretion does not contain [enough] compounds that irritate mice | N/A | [3] |
| *Aromobates nocturnus* | TLC, GC-MS, and thermospray mass spectrometry, and subcutaneous injection into white mice, all using methanol extract | no alkaloids detected and no effect on mice in extracts from 10 adult females | no alkaloids detected nor enough compounds that irritate mice, but we cannot rule out the occurrence of lipophilic alkaloids below detection threshold | N/A | [15] |
| *Aromobates nocturnus* | [^3^H]saxitoxin binding assay | no TTX detected in unknown number of individuals | unknown if lipophilic alkaloids present or absent | water-based extraction process would not retain high levels of lipophilic alkaloids, so cannot be used as evidence of presence or absence | [11] |
| *Colostethus imbricolus* | mouse bioassay and [^3^H]saxitoxin binding assay | no TTX detected in saxitoxin assay, but very marginal effects were noted in mouse assay, based on one skin sample | no direct evidence of lipophilic alkaloids, but potentially present | water-based extraction process would not retain high levels of lipophilic alkaloids, so cannot be used as evidence of presence or absence | [11,15] |
| *Colostethus imbricolus* | unknown (possibly GC-MS) | no lipophilic alkaloids detected in one skin sample | no alkaloids detected, but we cannot rule out the occurrence of lipophilic alkaloids below detection threshold | N/A | [15] |
| *Colostethus panamansis* (identified as “Colostethus inguinalis” in [11]) | feeding assay (alkaloid-dusted fruit flies) followed by GC-MS | no alkaloids detected in unknown number of individuals | no evidence for alkaloid accumulation after five weeks | N/A | [12] |
| *Colostethus panamansis* (identified as “Colostethus inguinalis” in [11]) | mouse bioassay, [^3^H]saxitoxin binding assay, HPLC, and TLC | TTX detected in three individuals | unknown if lipophilic alkaloids present or absent | water-based extraction process would not retain high levels of lipophilic alkaloids, so cannot be used as evidence of presence or absence | [11] |
| “*Colostethus* sp.” (identified as “Colostethus new species”, sympatric with *Aromobates nocturnus*, Trujillo, Venezuela in [11]) | either by mouse bioassay or by [^3^H]saxitoxin binding assay | no alkaloids detected (likely in one individual) | unknown if lipophilic alkaloids present or absent | water-based extraction process would not retain high levels of lipophilic alkaloids, so cannot be used as evidence of presence or absence | [11] |
| *Colostethus ucumari* | methanol extract (i.e., lipophilic compounds) caused minor agitation and twitching in mice, but aqueous extract (i.e., water-soluble compounds) caused gagging and some inactivity | non-toxic methanol extract (lipophilic compounds), non-toxic, aqueous extract (hydrophilic compounds), both from one individual (AMNH 104371) | evidence for presence of irritating lipophilic and hydrophilic compounds (possibly alkaloids) | N/A | [16] |
| *Colostethus ucumari* | GC-MS of methanol extract | small amounts of multiple lipophilic compounds not corresponding to known dendrobatid alkaloids from one individual (AMNH 104371) | lipophilic compounds present, potentially alkaloids | N/A | [16] |
| *Colostethus ucumari* | high-resolution GC-MS of methanol extract | only trace amount of one of lipophilic compounds detectable from 30-year-old methanol extract (AMNH 104371), which turned out to be artifact (2-benzothazolyl-N,N-dimethyl dithiocarbamate) | identity of lipophilic compounds remains unknown | N/A | [16] |
| *Ectopoglossus saxatilis* | GC-MS of methanol extract | no alkaloids detected in one individual | no alkaloids detected, but we cannot rule out the occurrence of lipophilic alkaloids below detection threshold | N/A | unpub. data reported in [17] |
| *Hyloxalus awa* | predator assay | naïve chicks readily ate unknown number of frogs | animal does not contain [enough] compounds that deter predators | this species was used as a control in predation experiments | [14] |
| *Hyloxalus azureiventris* | oral administration assay followed by GC-MS, feeding assay (alkaloid dusted fruit flies) followed by GC-MS | species sequestered four alkaloids dissolved in methanol-saline solution, but later studies found no sequestered alkaloids from dusted fruit flies, in unknown number of individuals | could sequester measurable levels of alkaloids, though likely inefficiently given the conflict between feeding and oral administration assays | N/A | unpub. data as reported in  [18] |
| *Hyloxalus azureiventris* | unknown (possibly GC-MS) | no alkaloids detected in unknown number of wild-caught individuals | no alkaloids detected, but we cannot rule out the occurrence of lipophilic alkaloids below detection threshold | N/A | unpub. data reported in [13] |
| *Hyloxalus chlorocraspedus* | unknown (possibly GC-MS) | no alkaloids detected in unknown number of individuals | no alkaloids detected, but we cannot rule out the occurrence of lipophilic alkaloids below detection threshold | N/A | unpub. data as reported in [19] |
| *Hyloxalus elachyhistus*  (identified as “*Hyloxalus infraguttatus*” in [12]) | either by mouse bioassay or by [^3^H]saxitoxin binding assay | no alkaloids detected (likely) in one individual | unknown if lipophilic alkaloids present or absent | water-based extraction process would not retain high levels of lipophilic alkaloids, so cannot be used as evidence of presence or absence | [11] |
| *Hyloxalus elachyhistus* (identified as “*Hyloxalus infraguttatus*” in [20]) | targeted GC-MS with nicotine standard followed by filtering against database of poison-frog alkaloids [21] | trace levels of diverse alkaloids, mostly indolizidines and histrionicotoxins, detected in study of nine individuals | presence of skin alkaloids | N/A | [20] |
| *Hyloxalus jhoncito* | GC-MS of methanol extract | no alkaloids detected in one individual | no alkaloids detected, but we cannot rule out the occurrence of lipophilic alkaloids below detection thresholds or not in the Daly database | N/A | unpub. data reported in [22] |
| *Hyloxalus nexipus* | TLC | spot observable in one of three individuals | spot on TLC plate suggests presence of skin metabolites (potentially alkaloids) | the negative control used in this experiment (*H. yasuni*) yielded a spot on the TLC plate but was assumed to lack alkaloids, which likely confounded the interpretation of results | [9] |
| *Hyloxalus sauli* | TLC | no spots detected in 10 individuals | no alkaloids detected, but image of TLC plate not available to verify interpretation | N/A | [2] |
| *Hyloxalus yasuni* (identified as “*Colostethus* sp. D” in [2]) | TLC | no spots detected in 22 individuals | no alkaloids detected, but image of TLC plate not available to verify interpretation | N/A | [2] |
| *Hyloxalus yasuni* (called “*Hyloxalus maculosus*” in [9]) | TLC | used as negative control, but spots were observable in both individuals (one spot in one individual, at least three in the other) | spots on TLC plate suggest presence of skin metabolites (potentially alkaloids) | used as negative control in [9] due to earlier TLC result for [2]) | [9] |
| *Hyloxalus vertebralis* | TLC | spot observable in two of five individuals | spots on TLC plate suggest presence of skin metabolites (potentially alkaloids) | the negative control used in this experiment (*H. yasuni*) yielded spots on the TLC plate but was assumed to lack alkaloids, which likely confounded the interpretation of results | [9] |
| *Leucostethus fraterdanieli*  (identified as “*Colostethus* sp. common nr Villa Maria, Caldas, Colombia in [11]) | either by mouse bioassay or by [^3^H]saxitoxin binding assay | no alkaloids detected (likely in one individual) | unknown if lipophilic alkaloids present or absent | water-based extraction process would not retain high levels of lipophilic alkaloids, so cannot be used as evidence of presence or absence | [11] |
| *Leucostethus fugax* | TLC | two spots observable in four individuals (one dark and one faint) and one spot observable in one individual | spots on TLC plate suggest presence of skin metabolites (potentially alkaloids) | the negative control used in this experiment (*H. yasuni*) yielded spots on the TLC plate but was assumed to lack alkaloids, which likely confounded the interpretation of results | [9] |
| *Leucostethus siapida* | GC-MS of methanol extract | no alkaloids detected in one individual | no alkaloids detected, but we cannot rule out the occurrence of lipophilic alkaloids below detection thresholds or not in the Daly database | N/A | unpub. data reported in [23] |
| *Mannophryne* cf. *collaris* (identified as “*Colostethus* new species, cf. *collaris*” Trujillo, Venezuela in [12]) | either by mouse bioassay or [^3^H]saxitoxin binding assay | no alkaloids detected (likely in one individual) | unknown if lipophilic alkaloids present or absent | water-based extraction process would not retain high levels of lipophilic alkaloids, so cannot be used as evidence of presence or absence | [11] |
| *Mannophryne riveroi* | either by mouse bioassay or by [^3^H]saxitoxin binding assay | no alkaloids detected (likely in one individual) | unknown if lipophilic alkaloids present or absent | water-based extraction process would not retain high levels of lipophilic alkaloids, so cannot be used as evidence of presence or absence | [11] |
| *Mannophryne trinitatis* | either by mouse bioassay or by [^3^H]saxitoxin binding assay | no alkaloids detected (likely in one individual) | unknown if lipophilic alkaloids present or absent | water-based extraction process would not retain high levels of lipophilic alkaloids, so cannot be used as evidence of presence or absence | [11] |
| *Paruwrobates erythromos* | GC-MS | three alkaloids detected across five individuals | presence of skin alkaloids | N/A | [1,24] |
| *Silverstoneia flotator* | 14-mo avian diet (regurgitate) study and herpetofauna census | 91 sympatric bird species do not consume *S. flotator* despite its being one of the two most common anurans | indirect evidence that animals may have compounds (possibly alkaloids) that deter predators | N/A | [25] |
| *Silverstoneia flotator* | feeding experiment with bat species (*Trachops cirrhosus*) | *Trachops cirrhosus* bats (from Barro Colorado Island, Panama) spit out unknown number of *S. flotator* individuals from Peninsula Gigante population (Panama) when hand-fed the frogs (field seasons 1979–81), and one bat even died as a result of frog consumption; it is highly unusual for this bat species to die after predation (Michael J. Ryan, pers. comm.) | indirect evidence that animals may have compounds (possibly alkaloids) that deter predators |  | (Michael J. Ryan, pers. comm.) |
| *Silverstoneia flotator* | GC-MS | no alkaloids detected in one individual | no alkaloids detected, but we cannot rule out the occurrence of lipophilic alkaloids below detection thresholds or not in the Daly database | the study tested ethanol in which whole animals were soaked after being collected | [6] |
| *Silverstoneia flotator* | unknown (possibly GC-MS) | no alkaloids detected in unknown number of individuals | no alkaloids detected, but we cannot rule out the occurrence of lipophilic alkaloids below detection threshold | N/A | Daly, unpub. data as reported in [6,25] |
| *Silverstoneia punctiventris* | head-space solid phase microextraction coupled to GC-MS (targeted method) | 13 compounds with alkaloid structures detected (including six from Daly’s poison-frog alkaloid database) across eight individuals | presence of six lipophilic alkaloids previously documented in poison frogs, seven other alkaloids | N/A | [26] |

**References**

1. Daly JW, Myers CW, Whittaker N. 1987 Further classification of skin alkaloids from neotropical poison frogs (Dendrobatidae), with a general survey of toxic/noxious substances in the Amphibia. *Toxicon* **25**, 1023–1095.

2. Darst CR, Menéndez-Guerrero PA, Coloma LA, Cannatella DC. 2005 Evolution of dietary specialization and chemical defense in poison frogs (Dendrobatidae): A comparative analysis. *Am. Nat.* **165**, 56–69.

3. Darst CR, Cummings ME, Cannatella DC. 2006 A mechanism for diversity in warning signals: Conspicuousness versus toxicity in poison frogs. *Proc. Natl. Acad. Sci. U. S. A.* **103**, 5852–5857.

4. Saporito RA, Grant T. 2018 Comment on Amézquita et al. (2017) “Conspicuousness, color resemblance, and toxicity in geographically diverging mimicry: The pan-Amazonian frog *Allobates femoralis*.” *Evolution* , 1009–1014.

5. Amézquita A, Ramos Ó, González MC, Rodríguez C, Medina I, Simões PI, Lima AP. 2017 Conspicuousness, color resemblance, and toxicity in geographically diverging mimicry: The pan-Amazonian frog *Allobates femoralis*. *Evolution* **71**, 1039–1050.

6. Mebs D, Yotsu-Yamashita M, Pogoda W, Vargas Alvarez J, Ernst R, Köhler G, Toennes SW. 2018 Lack of alkaloids and tetrodotoxin in the neotropical frogs *Allobates* spp. (Aromobatidae) and *Silverstoneia flotator* (Dendrobatidae). *Toxicon* **152**. (doi:10.1016/j.toxicon.2018.07.027)

7. Sanchez E *et al.* 2019 Transcriptomic signatures of experimental alkaloid consumption in a poison frog. *Genes*  **10**, 733.

8. Jeckel AM. 2021 Eficiência de sequestro e composição de alcaloides em rãs-de-veneno da família Dendrobatidae. Universidade de São Paulo. (doi:10.11606/t.41.2020.tde-08122020-132405)

9. Santos JC, Cannatella DC. 2011 Phenotypic integration emerges from aposematism and scale in poison frogs. *Proc. Natl. Acad. Sci. U. S. A.* **108**, 6175–6180.

10. Summers K, Clough ME. 2001 The evolution of coloration and toxicity in the poison frog family (Dendrobatidae). *Proc. Natl. Acad. Sci. U. S. A.* **98**, 6227–6232.

11. Daly JW, Gusovsky F, Myers CW, Yotsu-Yamashita M, Yasumoto T. 1994 First occurrence of tetrodotoxin in a dendrobatid frog (*Colostethus inguinalis*), with further reports for the bufonid genus *Atelopus*. *Toxicon* **32**, 279–285.

12. Daly JW, Secunda SI, Garraffo HM, Spande TF, Wisnieski A, Cover JF Jr. 1994 An uptake system for dietary alkaloids in poison frogs (Dendrobatidae). *Toxicon* **32**, 657–663.

13. Daly JW. 1998 Thirty years of discovering arthropod alkaloids in amphibian skins. *J. Nat. Prod.* **61**, 162–172.

14. Darst CR, Cummings ME. 2006 Predator learning favours mimicry of a less-toxic model in poison frogs. *Nature* **440**, 208–211.

15. Myers CW, Paolillo O. A, Daly JW. 1991 Discovery of a defensively malodorous and nocturnal frog in the family Dendrobatidae: Phylogenetic significance of a new genus and species from the Venezuelan Andes. *American Museum Novitates* **3002**.

16. Grant T. 2007 A new, toxic species of *Colostethus* (Anura: Dendrobatidae: Colostethinae) from the Cordillera Central of Colombia. *Zootaxa* **1555**, 39–51.

17. Grant T, Rada M, Anganoy-Criollo M, Batista A, Dias PH, Jeckel AM, Machado DJ, Rueda-Almonacid JV. 2017 Phylogenetic systematics of dart-poison frogs and their relatives revisited (Anura: Dendrobatoidea). *South Am. J. Herpetol.* **12**, S1–S90.

18. Saporito RA, Spande TF, Garraffo HM, Donnelly MA. 2009 Arthropod alkaloids in poison frogs: A review of the ‘Dietary Hypothesis.’ *Heterocycles* **79**, 277–297.

19. Grant T *et al.* 2006 Phylogenetic systematics of dart-poison frogs and their relatives (Amphibia: Athesphatanura: Dendrobatidae). *Bull. Am. Mus. Nat. Hist.* **299**, 1–262.

20. Moskowitz NA, Alvarez-Buylla A, Morrison CR, Chamba A, Rentería J, Tapia EE, Coloma LA, Donoso DA, O’Connell LA. 2022 Poison frog diet and chemical defense are influenced by availability and selectivity for ants. *bioRxiv*. , 2022.06.14.495949. (doi:10.1101/2022.06.14.495949)

21. Daly JW, Spande TF, Garraffo HM. 2005 Alkaloids from amphibian skin: A tabulation of over eight-hundred compounds. *J. Nat. Prod.* **68**, 1556–1575.

22. Anganoy-Criollo M, Viuche-Lozano A, Enciso-Calle MP, Bernal MH, Grant T. 2022 The enigmatic *Hyloxalus edwardsi* species group (Anura: Dendrobatidae): Phylogenetic position, a new species, and new putative morphological synapomorphies. *Herpetologica* **78**, 253–267.

23. Grant T, Bolívar-García W. 2021 A new species of *Leucostethus* (Anura, Dendrobatidae) from Gorgona Island, Colombia. *Zookeys* **1057**, 185–208.

24. Daly JW, Ware N, Saporito RA, Spande TF, Garraffo HM. 2009 N-methyldecahydroquinolines: An unexpected class of alkaloids from Amazonian poison frogs (Dendrobatidae). *J. Nat. Prod.* **72**, 1110–1114.

25. Poulin B, Lefebvre G, Ibáñez R, Jaramillo C, Hernández C, Stanley Rand A. 2001 Avian predation upon lizards and frogs in a neotropical forest understorey. *J. Trop. Ecol.* **17**, 21–40.

26. Gonzalez M, Palacios-Rodriguez P, Hernandez-Restrepo J, González-Santoro M, Amézquita A, Brunetti AE, Carazzone C. 2021 First characterization of toxic alkaloids and volatile organic compounds (VOCs) in the cryptic dendrobatid *Silverstoneia punctiventris*. *Front. Zool.* **18**, 39.
